# Comorbid Externalising Behaviour in AD/HD: Evidence for a Distinct Pathological Entity in Adolescence

**DOI:** 10.1371/journal.pone.0041407

**Published:** 2012-09-12

**Authors:** Sharnel Perera, David Crewther, Rodney Croft, Hannah Keage, Daniel Hermens, C. Richard Clark

**Affiliations:** 1 Brain Sciences Institute, Swinburne University of Technology, Melbourne, Victoria, Australia; 2 School of Psychology, University of Wollongong, Wollongong, New South Wales, Australia; 3 School of Psychology, Flinders University, Adelaide, South Australia, Australia; 4 Brain Dynamic Centre, University of Sydney, Sydney, New South Wales, Australia; 5 Cognitive Neuroscience Laboratory, School of Psychology, Social Work and Social Policy, University of South Australia, Adelaide, South Australia, Australia; The University of Queensland, Australia

## Abstract

While the profiling of subtypes of Attention Deficit Hyperactivity Disorder (AD/HD) have been the subject of considerable scrutiny, both psychometrically and psychophysiologically, little attention has been paid to the effect of diagnoses comorbid with AD/HD on such profiles. This is despite the greater than 80% prevalence of comorbidity under the DSM-IV-TR diagnostic definitions. Here we investigate the event related potential (ERP) and psychometric profiles of Controls, AD/HD, and comorbid AD/HD (particularly AD/HD+ODD/CD) groups on six neurocognitive tasks thought to probe the constructs of selective and sustained attention, response inhibition and executive function. Data from 29 parameters extracted from a child group (age range 6 to 12; 52 Controls and 64 AD/HD) and from an adolescent group (age range 13 to 17; 79 Controls and 88 AD/HD) were reduced via a Principal Components Analysis, the 6 significant eigenvectors then used as determinants of cluster membership via a Two-Step Cluster Analysis. Two clusters were found in the analysis of the adolescent age group - a cluster dominated by Control and AD/HD participants without comorbidity, while the second cluster was dominated by AD/HD participants with externalising comorbidity (largely oppositional defiant/conduct disorder ODD/CD). A similar segregation within the child age group was not found. Further analysis of these objectively determined clusters in terms of their clinical diagnoses indicates a significant effect of ODD/CD comorbidity on a concurrent AD/HD diagnosis. We conclude that comorbid externalising behaviour in AD/HD constitutes a distinct pathological entity in adolescence.

## Introduction

Although the Diagnostic and Statistical Manual of Mental Disorders, DSM-IV-TR [1] views Attention-Deficit/Hyperactivity Disorder (AD/HD) as a homogenous disorder, this view has been strongly contested. Previous research has repeatedly shown AD/HD to be heterogeneous in its presentation, genetics, severity, comorbidity, and treatment outcome [2], [3], [4], [5]. In a Swedish study [6] with children meeting the full criteria for AD/HD, 87% of their sample had at least one comorbid diagnosis and 67% had two or more comorbid diagnoses (of which oppositional defiant disorder and developmental coordination disorder were the most common). The multidimensional nature of the disorder is also suggested by the numerous theoretical models of aetiology and the variable global prevalence rates. Viewing AD/HD as a homogenous disorder may well account for negative or ambiguous findings in previous research, and it is for this reason that some authors have begun to investigate the existence of AD/HD subtypes or groups that are independent from those defined by the DSM-IV-TR, particularly in terms of comorbidity. In this regard, the World Health Organisation's International Classification of Diseases (ICD), which is used predominantly in Europe, defines comorbid combinations of disorders. Of particular interest is the ICD diagnosis of Hyperkinetic Conduct Disorder (HCD), which is the diagnostic equivalent to the combination of DSM-IV-TR's AD/HD and Oppositional Defiant Disorder (ODD) and/or Conduct Disorder (CD). Hence, the ICD considers AD/HD+ODD/CD to represent a distinct pathological entity, rather than a simple “combining of symptoms”. This view has gained some support in the previous AD/HD literature arguing for either the delineation of a distinct subtype of AD/HD comorbid with ODD/CD [7], or that AD/HD+ODD/CD should constitute a separate pathological entity altogether [8], similar to that adopted by the ICD.

The argument against a new *subtype* of AD/HD incorporating ODD/CD comorbidity has stemmed from research findings suggesting that AD/HD+ODD/CD represents a “hybrid” group where the symptomatology is additive [9], [10] and does not venture outside the realms of each disorder and hence does not constitute a distinct pathological entity. This finding was supported by a study by Rommelse et al [11] who found that while AD/HD+ODD/CD was a more severe form of AD/HD, it did not produce deficits in executive function (EF) and related motor components beyond the independent effects of AD/HD, and ODD/CD. In sharp contrast to this, Banaschewski et al [8] conducted a study utilising a cued Continuous Performance Task (CPT-AX) which found that the ‘hybrid’ concept was not able to account for the symptomatology of AD/HD+ODD/CD, and hence argued for the re-conceptualisation of AD/HD+ODD/CD as a distinct pathological entity. Though this debate has remained unresolved, it has fuelled research investigating the possible existence of AD/HD diagnostic groups that deviate from the DSM-IV-TR nomenclature.

Quantitative electroencephalography (QEEG) can play a pivotal role in documenting cerebral dysfunction in attention disordered individuals, and initial research has shown such results with AD/HD populations. A study by Chabot and Serfontein [12] found two distinct electrophysiological subtypes within their AD/HD population, both indicative of abnormal central nervous system arousal. Similarities in QEEG values were found between the AD/HD subtypes Inattentive (AD/HD-I) and Combined (AD/HD-C), suggesting comparability of underlying aetiology, and consequently providing a new perspective for subtype categorisation. Their study however, did not account for overt comorbidity such as Oppositional Defiant Disorder/Conduct Disorder (ODD/CD) within their AD/HD population. This may explain why later research by Clarke, Barry, McCarthy and Selikowitz [13] found conflicting results in terms of the type of QEEG similarities and differences in their AD/HD group. Clarke et al conducted a within-subtype analysis with children diagnosed as AD/HD-C (combined subtype) with no internalised (i.e. depression, anxiety, etc) or externalised (i.e. ODD/CD) comorbidity. Within this population, the authors isolated three distinct QEEG-defined subtypes associated with cortical hypoarousal, maturational lag, and cortical hyperarousal. An adjunct study focusing on AD/HD-I instead found very similar results with two QEEG profiles indicative of cortical hypoarousal, and maturational lag [14]. Event-related potentials (ERPs) from an Oddball task in the same AD/HD-I cohort showed only the early ERP negativity N1 to significantly differ between the QEEG-defined AD/HD-I subtypes - all other ERPs were comparable [15]. Two possible explanations ensue. It may be that the generation of cortical ERPs is primarily unaffected by underlying brain abnormalities, or secondly, both cortical hypoarousal and maturational lag are characterized by largely the same type of brain abnormalities or produce similar task-processing deficits. Since previous research has shown the EEG waveform to fluctuate and change in accordance with physical and mental activity [16], [17], it is reasonable to assume that any underlying EEG abnormality would be reflected in the task-related ERPs.

These studies suggest the existence of distinct AD/HD subtype groups that are independent of the behaviourally-defined DSM-IV-TR diagnostic criteria. To date, no investigation has been conducted into the ERP and psychometric profiles that may exist between/within Controls, AD/HD, and AD/HD+ODD/CD that lie outside the DSM-IV-TR diagnostic guidelines. This is of particular interest given the continuing debate regarding the classification of AD/HD+ODD/CD as a distinct pathological entity, in addition to the highly publicised symptom heterogeneity in AD/HD groups with and without comorbidity [3], [18] that can often result in behavioural and/or cognitive overlap between these groups. Therefore, the aim of this study is to ascertain whether ERP and psychometric performance profiles of AD/HD with and without ODD/CD comorbidity cluster into meaningful groups that suggest a divergence in the nomenclature of the DSM-IV-TR. A data-driven approach was adopted utilising six tasks probing the core deficits in AD/HD, including selective attention, sustained attention, hyperactivity and impulsivity, and executive function. These functions were examined both psychometrically and psychophysiologically (using event-related brain evoked potentials). Data-driven research has gained considerable support over the past decade as it can often generate unexpected findings that would not have been anticipated by hypothesis-driven research alone.

## Methods

### Participants

Electrophysiological and psychometric performance data was recorded from 152 AD/HD male participants and 131 healthy age-matched male Controls, within a study approved by the Swinburne Human Research Ethics Committee, with participants giving written informed consent (according with the Declaration of Helsinki). Although data from female AD/HD participants was obtained, the numbers between comorbid groups were insufficient to allow a reliable analysis by gender. Hence, only the data from the male participants are included in this study. Groups were subdivided into children aged 6–12 years, and adolescents aged 13–17 years (see Table 1).

**Table 1:** Average age and years of education for AD/HD and Control groups.

		AD/HD Subtype*	Subtype *n*	Total *N*	Mean Age (yr) (SD)	Years of Education Mean (SD)
**Children**	**AD/HD**	***I***	18	64	9.08 (1.52)	3.71 (1.60)
		***C***	42			
		***HI***	4			
	**Controls**			52	9.08 (1.52)	3.77 (1.59)
**Adolescents**	**AD/HD**	***I***	38	88	14.12 (1.43)	8.66 (1.53)
		***C***	48			
		***HI***	2			
	**Controls**			79	14.13 (1.43)	8.86 (1.58)

* *AD/HD subtypes: I = Inattentive, C = Combined, HI = Hyperactive/Impulsive.*

A one-way ANOVA showed no significant difference in age between AD/HD and Controls for either children [*F*(1,160)<0.01, *p* = .998], or adolescents [*F*(1,178)<0.01, *p* = .983], and no significant difference in educational level between AD/HD and Controls [children: *F*(1,160) = .050, *p* = .824; adolescents: *F*(1,178) = .746, *p* = .389]. Group size, age and level of education are shown below in Table 1.

All three subtypes of AD/HD (Inattentive: AD/HD-I, Combined: AD/HD-C, and Hyperactive/Impulsive: AD/HD-HI) were represented in the AD/HD population, although equal proportions could not be maintained. However, since the current investigation is not assessing subtype differences, an unequal representation of each subtype was not considered a significant limitation. A breakdown of AD/HD subtype numbers in each age group is provided in Table 1.

Participants were excluded if English was not their primary language, if they had a personal history of a physical brain injury, stroke, or other neurological disorder, or any serious medical condition related to the thyroid or heart or a history of cancer, unconsciousness resulting from a blow to the head within the last five years, a blood-borne illness, a severe impediment in vision (that could not be corrected, for example with glasses) such as colour vision, hearing or hand movement, a history of drug addiction, a history of heavy alcohol consumption, or a personal or familial genetic disorder. Participants (both AD/HD and Control) were also excluded if their IQ was below 80, as measured by the full-scale (WISC III) IQ or the Kaufman Brief Intelligence Test (K-BIT2) [19]. The Spot the Real Word test [20] was utilised to provide an ‘IQ estimate’ where WISC III or K-BIT2 information was unavailable. This test has shown a high correlation (*r* = 0.76) with the full-scale WAIS III IQ [21].

All AD/HD participants were referred by their respective clinician in the community, who confirmed AD/HD as the primary diagnosis prior to admission into the study. After referral, each AD/HD diagnosis was verified via the Diagnostic Interview for Children and Adolescents (DICA), completed by the parent or guardian at the time of testing. All AD/HD participants were either medication naïve at the time of testing, or had undergone a minimum 48-hour washout period prior to testing (20% of participants underwent a minimum 48-hour washout from methylphenidate, 8.24% from dexamphetamine, and 71.76% were medication naïve). AD/HD participants were excluded if any psychiatric disorder other than AD/HD was present (for example Tic Disorder, Autism Spectrum Disorder, etc). Where comorbidity was present, AD/HD was required as the primary diagnosis for inclusion in this study.

Exclusion criteria specific to Controls consisted of any personal or family history of AD/HD, or any other psychiatric disorder. To screen for any undiagnosed common psychiatric disorders within this cohort, the Somatic and Psychological Health Report 12 (SPHERE-12) [23], [24] was administered. All Controls identified as “SPHERE-12” cases were excluded.

Multi-site data collection was carried out from Australian laboratories located in Melbourne, Adelaide, and Sydney. Testing and practice protocols were identical between laboratories, conforming to the standards of the Brain Resource International Brain Database (BRID, Brain Resource Company), to ensure comparability of the data collected. Consistency between sites has been demonstrated [25], [26], [27], along with the reliability and validity of each psychophysiological and psychometric task contained within the BRID standard testing battery [26], [27], [28].

### AD/HD Comorbidity Profile

Details regarding comorbidity for each AD/HD participant was provided by their respective clinician and further corroborated by the DICA, completed by a parent or guardian at the time of testing. Comorbidities among the AD/HD population consisted of ODD/CD, Learning Disorder (LD), Anxiety (ANX), and Depression (DEP). Comorbidity in the child and adolescent AD/HD participants is shown below in Table 2.

**Table 2:** Comorbidity profile (number and percentage of sample) of the AD/HD children and adolescents.

	Externalising (ODD/CD)	Internalising* (LD/ANX/DEP)	None/Not known
**Children**	32 (50%)	11 (17%)	21 (33%)
**Adolescents**	35 (40%)	17 (19%)	36 (41%)

* *Includes three AD/HD participants with (1) social problems and high IQ, (2) fine motor delay, and (3) retardation; ‘LD’ = Learning Disorder, ‘ANX’ = Anxiety, ‘DEP’ = Depression.*

Where more than one comorbid disorder was present (12.63% of the AD/HD sample), grouping was based according to the presence or absence of ODD/CD. If ODD/CD was present as well as LD for example, the participant was grouped into the ODD/CD category. ‘AD/HD-NK’ will be used to denote AD/HD participants whose assessment of comorbidity by their paediatrician was negative at the time of interview, that is ‘no known’ comorbidity. This assessment was later confirmed at the time of testing via the DICA. Importantly, no participant in this group was diagnosed with ODD/CD. ‘AD/HD+INT’ will be used to denote AD/HD participants with internalising (Learning Disorder, Anxiety, Depression, etc) comorbidity.

A one-way ANOVA with Bonferroni post-hoc comparisons confirmed that there were no significant differences in age between these child [*F*(3, 158) = .123, *p* = .947], or adolescent groups [*F*(3, 176) = .746, *p* = .526].

Severity of behavioural pathology in the AD/HD cohort was measured via the Conners' Parent Rating Scale – Revised Long form (CPRS-R∶L) [22], with all subscales included: oppositional, cognitive problems/inattention, hyperactivity, anxious-shy, perfectionism, social problems, psychosomatic, AD/HD index, restless-impulsive, emotional lability, global index total, DSM-IV inattentive, DSM-IV hyperactive-impulsive, and DSM-IV symptoms total. Scores on each of these subscales were converted to *T*-scores prior to analysis via a one-way ANOVA with Bonferroni post-hoc comparisons. In children, AD/HD+INT scored higher on the anxious-shy subscale and emotional lability than AD/HD-NK (*p* = .003, *p* = .013 respectively), AD/HD+ODD/CD scored higher on social problems than either AD/HD+INT (*p* = .01) or AD/HD-NK (*p* = .005), AD/HD+INT scored higher than either AD/HD+ODD/CD or AD/HD-NK on inattentive score (*p*<.001 for both comparisons). In adolescents, AD/HD+ODD/CD scored higher on the oppositional subscale than either AD/HD-NK (*p* = .048) or AD/HD+INT (*p* = .005). AD/HD+ODD/CD also scored higher on hyperactivity than AD/HD+INT (*p* = .042) or AD/HD-NK (*p* = .036). AD/HD+ODD/CD scored higher than AD/HD+INT on the restless-impulsive subscale (*p* = .011), emotional lability (*p* = .009), and on the global index total (*p* = .003). On the latter subscale, AD/HD+ODD/CD also scored higher than AD/HD-NK (*p* = .019).

### Tasks and Procedure

All participants were seated in front of a computer screen in a light and sound attenuated room. Data from six psychophysiological and psychometric tasks were utilised in this investigation. These are listed below along with their respective domains of measurement shown in parenthesis.

- Auditory Oddball Task (selective attention)
- Continuous Performance “one-back” Task (sustained attention)
- Go/NoGo Task (response inhibition: RI)
- Verbal Interference Task (RI)
- Executive Maze Task (executive function: EF)
- Switching Of Attention Task (EF)

### Auditory Oddball Task

Participants were presented with high ‘target’ tones (1000 Hz) and low ‘standard’ tones (500 Hz) binaurally via headphones at 75 dB. The duration of each tone was 50 ms (including 5 ms rise/fall times), with an ISI of 1 s. All participants were instructed to respond via button-press to target tones only, with speed and accuracy equally stressed prior to task commencement. A short practice session was conducted to ensure task instructions had been understood.

A total of 340 tones were presented, (280 standard tones; 60 target tones). Tones were presented within a single block in a quasi-random order with no two target tones presented consecutively. Task duration was six minutes. ERPs were identified within the following component windows: N1 (70–120 ms), P2 (120–220 ms), N2 (120–300 ms), and P3 (220–550 ms). Indicators of behavioural performance consisted of Reaction Time (RT), Standard Reaction Time (SDRT), False Positives (FPs), and False Negatives (FNs).

### Continuous Performance Task (CPT)

The CPT utilised in this study consisted of a series of letters (B, C, D and G) which were presented one at a time on an otherwise blank computer screen. The duration of each letter was 200 ms, with an inter-stimulus interval (ISI) of 2.5 s. All participants were instructed to respond via button-press when the same letter appeared twice in a row, with speed and accuracy equally stressed prior to task commencement. A short practice session was allowed to ensure task instructions had been understood.

A total of 125 stimuli was presented: 85 background/non-target letters; 20 pseudo-randomly presented target letters (repetitions of the previous letter); and 20 distracter stimuli. The distracter stimulus consisted of a black and white checkerboard (with each black/white square being approximately 1×1 cm), which was randomly interleaved with the letter stimuli. All participants were instructed to ignore the checkerboards. Task duration was eight minutes. Indicators of psychometric performance on this task consisted of reaction time (RT) and its standard deviation (SDRT), false positive errors (FPs) and false negative errors (FNs).

### Go/NoGo Task (GNG)

The stimuli for the GNG task consisted of the word “PRESS” which was written in either green or red. A green PRESS was classified as the ‘Go’ stimulus, while the red PRESS was classified as the ‘No-Go’ stimulus. Each stimulus was presented on a computer screen for 500 ms, with an ISI of 1 s.

The word PRESS was presented on the computer screen in a pseudo-random order, a total of 28 times. A green (Go) PRESS was shown 21 of those times, while a red (No-Go) PRESS was shown 7 times. The green (Go) PRESS stimulus appeared 6 times consecutively at the beginning of the task so as to increase the perceived stimulus probability. This was followed immediately by a red (No-Go) PRESS stimulus. The remainder of the tasks consisted of random presentations of the Go and No-Go stimuli. Task duration was five minutes.

ERP component windows varied slightly in this task compared to the auditory Oddball task, and are defined as follows: N1 (95–170 ms), P2 (200–280 ms), N2 (220–350 ms), and P3 (300–450 ms). Indicators of psychometric performance consisted of RT, SDRT, FPs, and FNs.

### Verbal Interference Task (VIT)

The Verbal Interference Task (VIT) is a variant of the Stroop Colour-Word Test [29] which assesses the asymmetric pattern of interference control between colour-naming and word-reading [30]. The VIT differs qualitatively from the Stroop test only in the method of response; while the Stroop test requires a verbal response, the VIT employed a computerised (touch-screen) response system. The VIT utilised here comprises two components of progressive difficulty.

Colour words were presented on the touch-screen one at a time. In the first component, the participant was only required to identify the colour word by pressing the matching response word at the bottom of the touch-screen. In the second component, rather than identifying the colour word, the participant was required to identify the colour that the word was printed in, by pressing the matching response word. Both speed and accuracy were equally stressed in the task instructions and a short practise session was allowed to ensure these instructions had been understood. In both components, colour words would remain on the screen until the participant responded. The duration of each component was one minute.

Indicators of psychometric performance consisted of the number of correctly identified colour words from component one, and typeface colour from component two.

### Executive Maze Task (EM)

The Executive Maze task is a variant of the Austin Maze which primarily assesses visuo-spatial ability, memory, and learning, and also provides secondary insight into planning, error utilisation, and working memory abilities [31].

The Executive Maze task was presented on a computer screen as a grid (8×8 matrix) of circles. The objective of this task is to find and remember a hidden path through the grid. Participants were required to use a directional button box in order to navigate their way through the grid and find the hidden path through trial and error. Correct moves were denoted by a green tick at the bottom of the screen and accompanied by a tone, whereas incorrect moves were denoted by a red cross at the bottom of the screen and accompanied by a, lower-pitched tone. When the participant was able to complete the maze without error twice consecutively, the task concluded. Each participant was given no longer than seven minutes to reach this goal, after which this task was discontinued. Indicators of psychometric performance consisted of the total number of errors made, and the total time taken to complete the task.

### Switching Of Attention Task (SOAT)

The Switching of Attention Task (SOAT) is a variant of the Trail Making Test [32] and assesses general attentional functioning and executive function (planning, and switching of attention), visuomotor tracking, and motor speed. The SOAT comprises two components or ‘trails’ of differing difficulty levels; the first trail measures the basic ability to maintain attentional focus on a simple task, while the more challenging second trail measures the ability to alternate attention between two simple mental sets.

In the first trail, participants are presented with 25 numbered circles in ascending order (that is, 1 – 2 – 3, etc). These circles are scattered in a fixed pseudo-random order on the touch-screen and the participant is required to identify each circle in ascending numerical sequence by touching the appropriate circle on the touch screen. The second trail involves the identification of both numbers and letters in ascending but alternating order (that is, 1 – A – 2 – B – 3 – C, etc). The numbers 1–13 and the letters A–L are presented in circles, again in a fixed pseudo-random order on the touch-screen. Indicators of psychometric performance consisted of the time taken to complete each trail. Electrophysiological data was not collected during this task.

### EEG Acquisition

Data was acquired from 32 channels including 26 scalp sites: Fp1, Fp2, F7, F3, Fz, F4, F8, FC3, FCz, FC4, T3, C3, Cz, C4, T4, CP3, CPz, CP4, T5, P3, Pz, P4, T6, O1, Oz, and O2 (NuAmps, Neuroscan, Melbourne, Australia; 10–20 International System). Impedance was below 5 kΩ at the beginning of testing. Data were recorded relative to the virtual ground and re-referenced offline to linked mastoids. Horizontal eye-movements were recorded with electrodes placed 1.5 cm lateral to the outer canthus of each eye. Vertical eye movements were recorded with electrodes placed 3 mm above the middle of the left eyebrow and 1.5 cm below the middle of the left bottom eye-lid. A continuous acquisition system was employed and data was EOG-corrected offline [33]. The sampling rate was 500 Hz. Individual single-trial epochs were filtered with a low-pass Tukey (cosine taper to 35 Hz) filter. The single trials were then averaged to form conventional ERPs to deviants, from which N1, N2, P2 and P3 amplitudes and latencies were derived according to the component windows specified above.

#### Spatial averaging of EEG data

In order to maximise the amount of meaningful data included in the analysis, ERPs were averaged across multiple scalp sites according to topographic location and areas of maximal activation (see Table 3), as is typical of studies that incorporate a large amount of ERP data [34], [35], [36]. This was done regardless of cognitive paradigm.

**Table 3:** Spatial averaging of ERPs.

Topographic Location	Scalp Sites Averaged
Fronto-Central N1	Fp1, Fp2, F7, F3, Fz, F4, F8, FC3, FCz, FC4
Fronto-Central N2	Fp1, Fp2, F7, F3, Fz, F4, F8, FC3, FCz, FC4
Central P2	T3, C3, Cz, C4, T4, CP3, CPz, CP4
Centro-Parietal P3	CP3, CPz, CP4, T5, P3, Pz, P4, T6

### Analysis

A total of 29 variables were selected for inclusion in a Principal components analysis (PCA) (see Table 4). Reaction times (RT) were established for oddball and CPT tasks, and ERP data was selected from Oddball and Go-NoGo tasks.

**Table 4:** Final 29 variables included in the Principal components analysis.

Variable	Task	Underlying Construct
***Psychometric:***		
Incongruent Trial Score	VIT	RI
Incongruent Error Score	VIT	RI
Trail Completion Difference*	SOAT	EF
Maze Trial Time*	EM	visual information processing/task performance
Perseverative Errors	EM	visual information processing/task performance
Non-Perseverative Errors	EM	visual information processing/task performance
Path Learning Time*	EM	visual information processing/task performance
RT	Oddball	selective attention
FNs	Oddball	selective attention
FPs	GNG	RI
RT	CPT	sustained attention
FNs	CPT	sustained attention
Total FPs	CPT/Oddball	hyperactivity/impulsivity
***Electrophysiological*** # ***:***		
N1A, N1L	Oddball	orienting of attention
P2A, P2L	Oddball	automatic inhibition
N2A, N2L	Oddball	inhibitory/mismatch process
P3A, P3L	Oddball	complex information processing
N1A, N1L	GNG	orienting of attention
P2A, P2L	GNG	automatic inhibition
N2A, N2L	GNG	inhibitory/mismatch process
P3A, P3L	GNG	complex information processing

* *‘Trail Completion Difference’ was measured as the difference in completion times between the two trails of the SOAT; ‘Maze Trial Time’ is the time taken to complete the trial twice consecutively without error; ‘Path Learning Time’ is the time taken to learn the path prior to completing the trial twice consecutively without error (i.e. the time taken from the start of the first trial till the end of the last trial with one or more errors).*

# *The letter ‘A’ or ‘L’ is added to the end of each ERP component to denote amplitude (A) or latency (L), for example ‘N1A’ denotes fronto-central N1 amplitude.*

### Statistical Analysis

Prior to statistical analysis, square root transformations were performed on all error scores (FPs, and FNs) due to their skewed distributions. Analysis of data in this study was two-fold. Firstly, psychometric performance, and amplitude and latency ERP variables from the six tasks were incorporated into a Principal Components Analysis (PCA) in order to reduce the amount of data. Secondly, a Two-Step Cluster Analysis using the PCA-derived factors was conducted using a log-likelihood distance measure and the Schwarz's Bayesian Clustering Criterion (BIC). The Cluster Analysis was run for both children and adolescents, with no number of clusters specified *a priori*. Bonferroni corrections were applied.

Since variables with larger values can have a stronger impact on clustering than those with smaller values [37], all of the PCA-derived factors were automatically standardised as z-scores (*x* = 0, *SD* = 1) prior to analysis, as part of the two-step Cluster process.

Significant differences between clusters in each age group were determined via Mann-Whitney U Tests due to skewed distributions in the PCA-derived factors. Permutation testing on group centroid distances in Z-space of the most significant predictors was also carried out.

## Results

### Principal Components Analysis

The factorability of these 29 variables used (Table 4) was supported by both the Kaiser-Meyer-Oklin value (.811) which exceeded the recommended threshold of .60 [38], and by a significant Bartlett's Test of Sphericity (*p*<.001) [39]. Nine components were found with eigenvalues (proportional to the total variance explained by that eigenvector) greater than one. Since components in the PCA were standardised to a variance of 1, only eigenvalues >1 were retained. However, following a Parallel Analysis [40] with 100 randomly generated replications of the same dataset matrix, only six Principal components were finally retained (Parallel Analysis was conducted using the Monte Carlo PCA for Parallel Analysis computer software [41]).

An exploratory factor analysis was then carried out. Given that each PCA component is considered to represent a different facet of attention and cognition, an oblique rotational method was employed. The six Principal components were rotated using a Promax rotation. The pattern matrix from this rotation is shown in Table 5.

**Table 5:** Promax rotated pattern matrix - six eigenvector solution from PCA.

	EigenV 1	EigenV 2	EigenV 3	EigenV 4	EigenV 5	EigenV 6
P3L (GNG)	.739					
N2L (GNG)	.721					
P3L (Oddball)	.652					
N1A (GNG)	−.593					
EF(SOAT)	.539					
P2L (GNG)	.528					
RT (Oddball)	.493					
Incong. Trial Score	−.451					
N1L (GNG)	.449					.324
RT (CPT)	.409					
Persev. Errors		.923				
Non-Persev. Errors		.896				
Time per Trial		.728				
Path Learning Time	.349	.524				
Total FPs			.788			
FNs (Oddball)			.661			
FPs (GNG)			.599			
FNs (CPT)			.566			
Incong. Error Score			.531			
P2L (Oddball)				.873		
N1L (Oddball)				.784		
N2L (Oddball)	.366			.655		
N1A (Oddball)				−.582		.404
P2A (GNG)	.551				.767	
N2A (GNG)					.628	
P3A (GNG)			−.327		.525	
P3A (Oddball)					.487	.307
P2A (Oddball)						.815
N2A (Oddball)	−.484		.384			.582

Each of the six components identified via the PCA comprised of a range of variables originating from different tasks, which renders a sensible naming difficult. Thus, for each component, the variables that had the highest loadings will be taken as the best representative of any underlying construct(s) [42]. According to Hair, Anderson, Tatham and Black [43], a loading of 0.6 or above is considered as “high”, and a loading of 0.7 indicates that roughly half of the variance in that factor is accounted for by that variable.

The largest number of task variables were grouped together to form Factor 1. Of these variables, three had loadings at or above 0.6 these were the ERP components P3 and N2 latency from the GNG task, along with P3 latency from the Oddball task. The N2 ERP component has previously been found to be a reliable marker of the inhibitory process [44], [45], [46], in addition to stimulus discrimination or the ‘mismatch detection’ process [47], [48]. Given this, it appears that Factor 1 is representative of complex processing related to task difficulty. Since this Factor is largely comprised of ERP component latency and RT variables, higher scores would be indicative of greater impairment.

Factor 2 was wholly comprised of variables from the Executive Maze task with 3 variables possessing factor loadings >0.7 (Preservative Errors, Non-Preservative Errors, and Time per Trial), suggestive of deficits in visuo-spatial abilities as previously identified in AD/HD [43], [49] and AD/HD+ODD/CD [50] cohorts.

The variables that comprised Factor 3 were error-related, with only two (Total FPs and FNs from the Oddball task) having a factor loading at or higher than 0.6 (though with FPs from the GNG task giving a factor loading = .599). Together they could be described as relating to error monitoring.

Factor 4 was wholly comprised of ERP components derived from the auditory Oddball task; four in total with three being latency ERPs. Out of these four variables, two possessed factor loadings above 0.7 (P2L and N1L), and one had a factor loading above .6 (N2L). Previously, these ERPs have been reported as reflective of the initial orienting of attention (N1) [47], [48], [51], [52], and the automatic inhibition of irrelevant stimuli (P2, N2) [44], [46], [47], [48], [53]. Note that in a fashion similar to Factor 1, higher scores in Factors 2, 3, and 4 reflect greater impairment.

Factor 5 showed major contributions from four variables, mostly derived from the visual GNG task. Of the four variables, two had factor loadings ≥0.6 (P2A and N2A), with one of out the two having a factor loading above >.7 (P2A). Given that the ERP components P2 and N2 have been suggested to reflect facets of the inhibitory process, this Factor may therefore be interpreted as corresponding to response inhibition [54], [55].

Factor 6 possessed only one variable with a factor loading >.6, though another variable did approach this threshold (N2A: factor loading = .582). As both ERP components here were derived from the auditory Oddball task, this factor may be representative of an auditory selective attention process.

### Cluster Analysis

The six rotated Factors obtained via the PCA were then subjected to a Cluster Analysis to investigate the possible presence of AD/HD groups that differ from those defined in the DSM-IV-TR.

### Adolescent group

**Figure 1:**
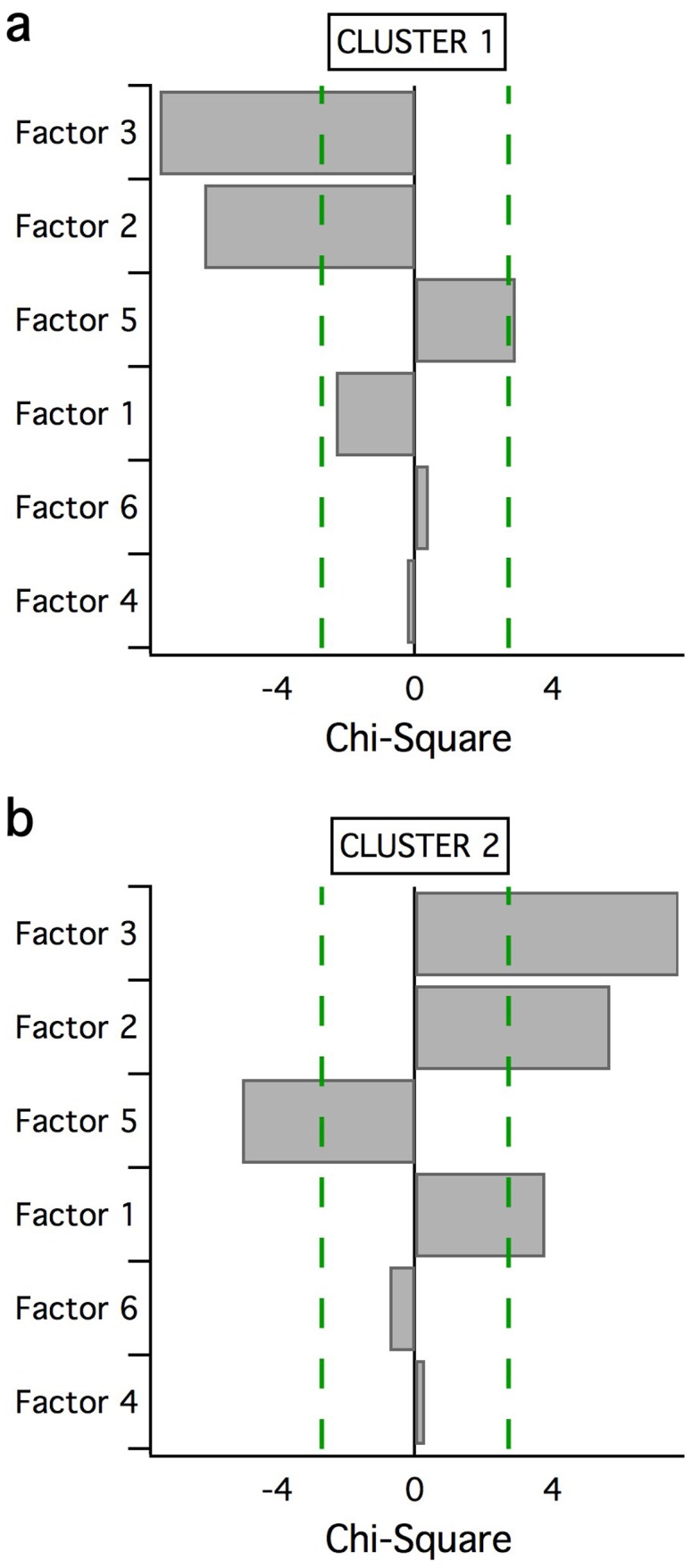
Variable-wise importance charts. Chi-square values for each of the six factors are shown with significant factors (those that exceeded the Critical Value indicated by the dashed lines) highlighted for (a) Cluster 1, and (b) Cluster 2, in descending order.

In the adolescent analysis, two clusters were identified with 113 participants in the first cluster (Cluster 1), and 54 participants in the second cluster (Cluster 2). Between the two adolescent clusters, Cluster 2 appears to be more of a ‘Clinical’ group due to the comparatively greater populations of AD/HD+ODD/CD and AD/HD+INT (internalising) than in Cluster 1 which is predominately comprised of Controls (see Table 6 and Figure 1).

**Table 6:** The two clusters produced from the adolescent cluster analysis, and the percentage distribution of Clinical and Control participants in each cluster.

Cluster 1	*N*	Cluster %	Cluster 2	*N*	Cluster %
Controls	71	63.7%	Controls	7	13.0%
AD/HD+ODD/CD	11	9.7%	AD/HD+ODD/CD	24	44.4%
AD/HD+INT	7	6.2%	AD/HD+INT	10	18.5%
AD/HD−NK	23	20.4%	AD/HD−NK	13	24.1%

*Comorbidities ODD/CD: Oppositional Defiant Disorder/Conduct Disorder; INT: internalising comorbidities; NK: no known comorbidity.*

**Figure 2:**
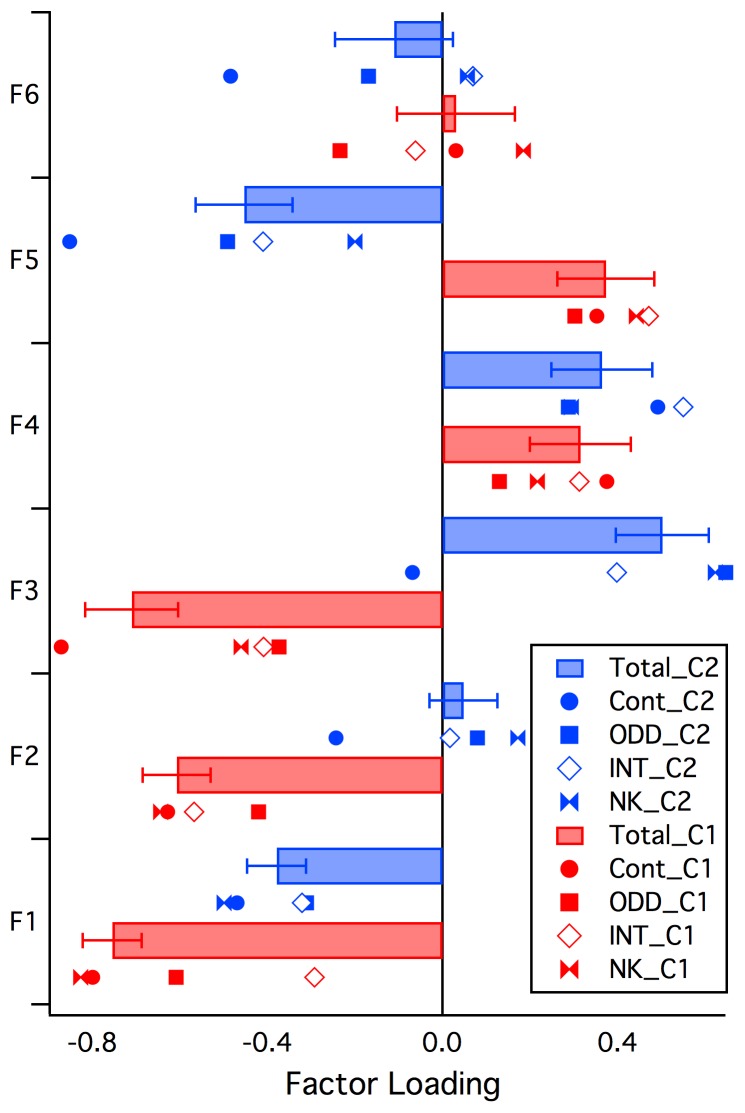
Mean Factor loadings and standard errors of the adolescent Clusters 1 and 2 (Total C1, Total C2) for each of the six factors (F1–F6). In addition the mean Factor scores for each of the Comorbidity subcategories are plotted (Control: Cont; AD/HD+ODD/CD: ODD; Internalising: INT; AD/HD-NK: NK).

Of the six factors, Factors 2, 3, and 5 all significantly (with Bonferroni corrections) contributed to defining Clusters 1 and 2. Factors 2 and 3 contributed significantly more to Cluster 2 than Cluster 1, and Factor 1 was more prominent in Cluster 2 compared to Cluster 1 (see Figure 2). As discussed earlier, Factor 1 is thought to represent complex processing related to task difficulty, and Factors 2 and 3 are thought to be indicative of task performance deficits, with Factor 2 being more specific to visuo-spatial processing. Therefore, it appears that Cluster 2 displayed more task performance deficits, and impaired complex processing related to task difficulty, compared to Cluster 1.

Of particular interest was the greater number of AD/HD-NK adolescents in Cluster 1 than Cluster 2. To see whether this cluster distribution was an effect of AD/HD subtype, a Chi-Square Test was conducted post-hoc (with AD/HD-HI excluded from this analysis since there were only two adolescents in total). The difference in AD/HD subtype distributions between Clusters 1 and 2 did not reach significance (*χ* ^2^ = 2.096, *p* = .148). Therefore, the characteristics of each adolescent Cluster is independent of AD/HD subtype. A second Chi-Square test was conducted to verify that comorbidity significantly differed between the two adolescent Clusters; this was confirmed: *χ* ^2^ = 46.587, *p*<.001. A one-way ANOVA was also conducted to confirm that cluster distribution was not an effect of intelligence; no significant difference in IQ estimates was found between Clusters 1 and 2 [*F*(1, 166) = .372, *p* = .543].

**Figure 3:**
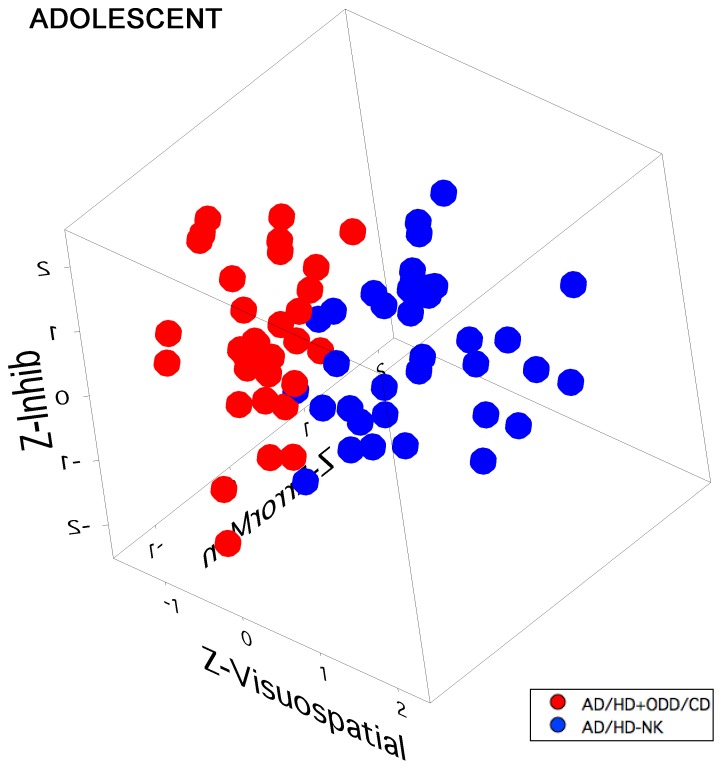
3D scatter plot of Adolescent AD/HD+ODD/CD (red dots) and AD/HD without comorbid diagnosis (blue dots) plotted on axes of the most significant factors (2,3 and 5) from the PCA analysis, scaled as Z-scores (Z-Error Monitoring, Z-Inhibitory processing, Z-Visuospatial learning, respectively).

Importantly, the Z-score data from the three most predicative factors for cluster membership (Eigenvectors 2, 3 and 5) were subjected to a permutation testing routine to test the hypothesis that comorbid diagnosis of AD/HD+ODD/CD would show an objective separation from the group comprising AD/HD without a diagnosis of ODD/CD (a comparison of AD/HD with Externalising comorbidity with other AD/HD participants (with Internalising or no comorbidity)). Visualization of the data via a 3D scatter plot shows a clear separation of the two groups (see Figure 3, and the movie in Supplementary Movie S1).

The Pythagorean distance between centroids of the two groups of points was then calculated. A permutation test was constructed using LabVIEW (National Instruments, Austin USA) based on the null hypothesis that all of the points derive from one population and were thus randomly selected into two groups, of the same sizes as the experimental groups, 10000 times, and each time the Pythagorean distance between centroids in Z-space was calculated. The experimental datum (1.0856) ranked 4^th^ highest, resulting in an equivalent (two-tailed) probability of *p*<.001. Testing of the other combinations of clinical AD/HD comorbid diagnoses did not result in significant separation.

### Child group

**Figure 4:**
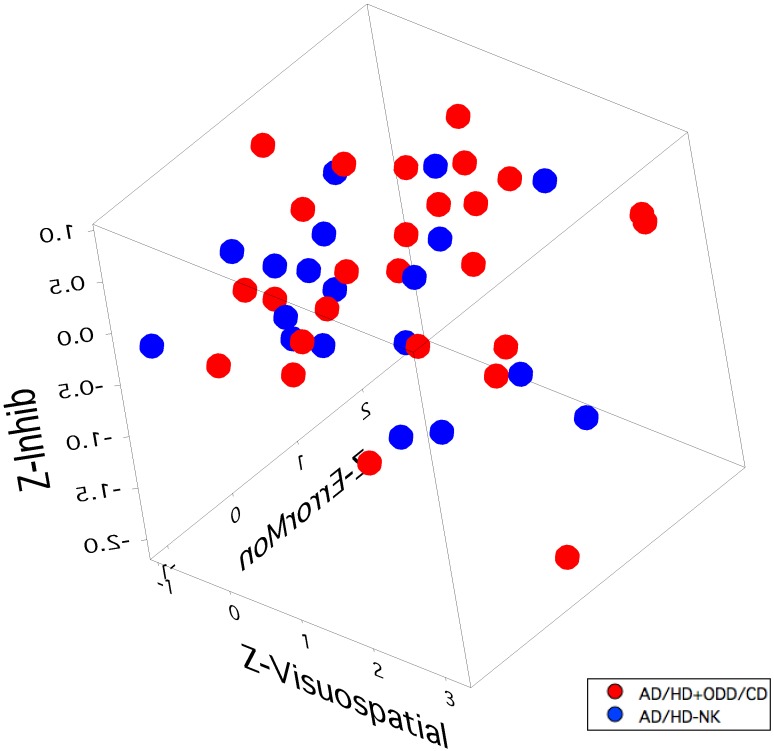
3D scatter plot of Child AD/HD+ODD/CD (red dots) and AD/HD without comorbid diagnosis (blue dots) plotted on axes of the same factors (2,3 and 5) predictive of clustering in the adolescent analysis, scaled as Z-scores (Z-Error Monitoring, Z-Inhibitory processing, Z-Visuospatial learning, respectively).

Only one cluster was found in the child analysis which comprised all 116 Clinical and Control children. The scores on each of the six factors from the PCA analysis did not display any pattern that could segregate the children into more than one cluster. It appears that that the variance in the six factors was too great to result in a significant difference between children from the Clinical and Control groups. This view is borne out by visualization of the 3-factor plot (factors 2, 3 5) for children with AD/HD+ODD/CD and those with AD/HD without known comorbidity (see Figure 4, and the movie in Supplementary Movie S2).

## Discussion

The aim of this study was to investigate whether the child and adolescent Clinical and Control populations could be clustered into meaningful groups divergent from that defined by the DSM-IV-TR. This clustering was based on the six factors from a Principal Components Analysis of 29 data variables derived from a battery of six neurocognitive tasks. The relative impact of ODD/CD comorbidity in AD/HD was also investigated. The results showed a single collective cluster in the child group, and two clusters in the adolescent group which were suggestive of ‘clinical’, and ‘normal/sub-clinical’ populations.

No significant factors were found in the child analysis. In adolescence, three factors (2, 3, and 5) dictated cluster membership more so than any of the other factors. Both Factors 2 and 3 are thought to be indicative of task performance, with Factor 2 being more specific to visuo-spatial processing. Factor 5 was thought to reflect response inhibition specific to visual information processing. Factor 1 was also found to significantly discriminate Cluster 2 from Cluster 1, though not vice versa. Factors 2 and 3, which are both thought to be related to task performance, were found to be the two most prominent factors in this analysis. This is unsurprising given that previous research has found task performance to be significantly more impaired in AD/HD populations, particularly when inhibitory tasks are involved [46], [56], [57], [58], [59], [60], [61]. Therefore, it was also unsurprising to find that Cluster 2, which had the larger Clinical population, displayed substantially more impairment as indexed by these two factors than Cluster 1. This finding is strengthened by the fact that greater impairment in complex processing relating to task difficulty was a defining characteristic of Cluster 2, rather than Cluster 1. The significant differences in Factor 5 were also strongly indicative of comparatively more impairment in Cluster 2 than Cluster 1.

In the child analysis, only one cluster was found which included all 116 Clinical and Control children. Although AD/HD is typically first diagnosed in childhood, this is also the time when behaviour and development (both physical and cognitive) is the most fluid; any model of “misbehaviour” may be difficult to apply to young cohorts as it may only be representative of a transient phase in development. As a result, any variable dependent on such factors is likely to be applicable only to a more discrete point in time in the child's development. This highlights the massive scope for variability, which was evident in each of the six factors. Of the 116 Clinical and Control children, none of the data on the six factors showed any form of homogeneity that would allow cluster formation due to the substantial amount of variability. Therefore, this result supports the widely held contention in AD/HD research that the overwhelming symptom heterogeneity which is consistently found in AD/HD populations appears to be at its most pronounced in childhood. Given this, a more individualistic approach to diagnosis of disruptive behaviour disorders, such as AD/HD, in childhood is encouraged, as such heterogeneity can impede effective treatment regimes if a generalised approach is adopted at this age.

Comorbid subgroups in childhood AD/HD did not cluster into meaningful groups divergent from the core disorder of AD/HD or from Controls, suggesting that overt and covert comorbidity can manifest as highly variable symptomatology that is not dissimilar between groups. Previous research has shown comorbidity such as ODD/CD to be more prevalent in older AD/HD cohorts [62], therefore, AD/HD children diagnosed with comorbid ODD/CD may be more representative of a prodromal comorbid group that are less symptomatic and less impaired than their older counterparts. This result therefore challenges the reliability of comorbid ODD/CD diagnoses in AD/HD children aged 6–12 years, and highlights the need for age-appropriate diagnostic criteria.

The adolescent analysis produced quite different results to that of the children. Two clusters were found; Cluster 1 which resembled a more ‘normal’ or ‘sub-clinical’ group, and Cluster 2 which clearly represented a more ‘clinical’ group. This interpretation was primarily fuelled by the population distributions of the Controls, AD/HD+INT, and AD/HD+ODD/CD. Almost two thirds of the entire Cluster 1 population were Controls, while almost half of Cluster 2 was AD/HD+ODD/CD. There were also slightly more AD/HD+INT adolescents in Cluster 2 than there were in Cluster 1. Given this, it appears that ODD/CD comorbidity in AD/HD is a primary factor in distinguishing behavioural and/or attentional dysfunction against Controls and hence may bias the diagnosis of AD/HD in adolescents. In the absence of such comorbidity, AD/HD-NK displayed a more varied result with 64% of the total population grouped into Cluster 1 with the bulk of the Controls, and 36% grouped into the more ‘clinical’ Cluster 2. Firstly, this suggests some overlap in neurocognitive performance, or some confusion of behavioural diagnosis. Such an overlap can be interpreted as representing dimensional impairment if members of the AD/HD-NK group in Cluster 2 do in fact possess sub-clinical levels of ODD/CD symptomatology. Future research is needed to clarify the dimensional nature of symptomatology and symptom severity between AD/HD comorbid groups. Secondly, the AD/HD-NK in Cluster 1 may represent the gradual dissipation of overt symptoms with age [63], [64], [65], [66] and hence, the general decrease in dysfunction and symptom severity. Both of these explanations may be concurrently valid.

The present results in terms of AD/HD-NK can be seen as reminiscent of previous QEEG findings which have typically shown two groups within the AD/HD subtypes that are independent from the DSM-IV-TR definition [13], [14], [67]. With the two cluster-defined AD/HD-NK groups, Cluster 2 clearly displayed more impairment than Cluster 1, which was not found to be an effect of AD/HD subtype. In the previous research also, two groups (cortical hypoarousal and maturational lag) were found, though analyses did not reveal any significant differences to indicate a more impaired group [15]. The concepts of maturational lag and cortical hypoarousal have repeatedly been applied to AD/HD populations in the previous literature [68], [69], [70], [71], with positive results suggesting both theories are equally valid, however some authors argue that AD/HD subjects display deviant maturation, rather than maturational lag *per se* [72]. Cortical hypoarousal in particular has recently been linked to inhibition [69]. It is also possible that the two theories are linked, rather than occurring in parallel, that is, one might act as a catalyst for the other. It is difficult to declare that the two AD/HD-NK groups found here displayed signs of maturational lag or cortical hypoarousal as the present results did not incorporate an analysis of quantitative EEG or imaging data. However, an early study linked developmental immaturity to persistent and extreme overactivity [73], suggesting that clinical levels of hyperactivity and impulsivity were indicative of a maturational lag. Given this finding, it can be reasoned that the more impaired AD/HD-NK in cluster 2 may have displayed a maturational lag compared to the less impaired AD/HD-NK cohort in cluster 1. This contention is based on the type of Factors that were most successful in identifying task-defined symptom severity, and subsequent cluster membership; Factors 2, 3 and 5 represent components of attention, learning, and inhibition, all of which are strongly influenced by hyperactivity and impulsivity.

Hyperactive and impulsive symptoms have consistently been linked to ODD/CD comorbidity, and greater overall symptom severity in AD/HD samples [74], [75], [76], [77], [78], [79], [80]; a study by Decker et al. [81] for example, found comorbid CD is more likely to be diagnosed in AD/HD subtypes with hyperactive/impulsive symptoms than inattentive symptoms. Given this, the primary distinguishing characteristic between the adolescent clusters 1 and 2 is likely to be task-defined hyperactivity and/or impulsivity, as captured by Factors 2, 3 and 5, which appear to be most pronounced in AD/HD+ODD/CD adolescents.

Task performance as dictated by these three factors also illustrated a clear distinction between AD/HD-NK and AD/HD+ODD/CD adolescents, suggesting a significant divergence in task-defined symptom severity. Previous research on comorbid AD/HD has repeatedly shown AD/HD+ODD/CD to display significantly greater symptom severity compared to AD/HD-alone [62], [77], [82]. This result supports previous claims that AD/HD+ODD/CD may constitute a distinct pathological entity [8] rather than a ‘hybrid’ group. A hybrid group would be expected to display a noticeable overlap in task performance scores with AD/HD+ODD/CD, suggesting a dimensional increase in symptom severity, however this did not appear to be the case here. Rather, it appears that task-defined symptom severity in AD/HD+ODD/CD adolescents is beyond that defined under the AD/HD-NK umbrella.

From the results found here, the most intriguing was the lack of any Clinical/Control cluster formations in the child analysis. In sharp contrast to the adolescent results, the six factors did not show any distinguishable pattern between any of the Clinical or Control groups and as a result, all of the children were clustered together. This finding could partly be accounted for by the inherent heterogeneity in AD/HD, however similar variability appeared to be present in the Control children also. This suggests that symptomatology and symptom severity in AD/HD exists on a dimensional scale that stems from ‘normal’ cognitive and behavioural function as seen in the Controls, rather than an arbitrary counting of symptoms deemed to be abnormal or maladaptive as per the DSM-IV-TR definition. Given that both physical and cognitive development is at its most fluid state in childhood, the single cluster result found for this age group is contextually unsurprising.

Overall, the adolescent clusters differed primarily in terms of task-related hyperactivity and/or impulsivity as defined by error rate (Factors 2 and 3) and visual response inhibition (Factor 5). The results obtained with these Factors suggest that measures of hyperactivity/impulsivity and visual response inhibition may serve as diagnostic aids in a clinical setting, or as profiling anchors in future research. These factors indexed greater impairment, particularly when ODD/CD comorbidity was present in AD/HD. Given this finding, the present results support the idea that AD/HD+ODD/CD can be distinguished on a dimensional scale from AD/HD-NK in adolescence. Hence, it may prove beneficial for comorbidity such as ODD/CD to be incorporated into the diagnostic definition of AD/HD and consequently into the diagnostic process, particularly when AD/HD progresses from childhood into adolescence. Such a stance has already been adopted in the ICD, where a distinct diagnosis of Hyperkinetic Conduct Disorder (HCD) is made for AD/HD+ODD/CD [83]. The question then arises as to whether or not AD/HD+ODD/CD should be defined as a distinct pathological entity in the forthcoming DSM-V. Although the affirmative has been argued by Banaschewski et al [8], others have argued that AD/HD+ODD/CD is more of a ‘hybrid’ group characterised by a greater severity of the *same* symptomatic domains [9], [10], a contention supported by Rommelse et al [11] who described AD/HD+ODD/CD symptomatology as “more of the same” (p. 802) rather than a phenotypically distinct subtype. The results from this study indicate that ODD/CD comorbidity has a significant impact on the neurocognitive performance of adolescents diagnosed with AD/HD and hence supports a revision of the current AD/HD nomenclature to allow AD/HD+OD/CD to be seen as a distinct pathological entity, however this appears to be valid only in adolescence; there does not appear to be a similar pattern of results supportive of such nomenclature in childhood. Rather, childhood diagnosis would benefit from a dimensional approach to symptomatology and symptom severity.

## Supporting Information

Movie S1 **Movie showing an orbital view of the 3D scatter plot of Adolescent AD/HD+ODD/CD (red dots) and AD/HD without comorbid diagnosis (blue dots) plotted on the Z-Error Monitoring, Z-Inhibitory processing and Z-Visuospatial learning axes.** A clear separation of the red and blue dots is seen.
(MOV)

Movie S2 **Movie showing an orbital view of the 3D scatter plot of Child AD/HD+ODD/CD (red dots) and AD/HD without comorbid diagnosis (blue dots) plotted on the Z-Error Monitoring, Z-Inhibitory processing and Z-Visuospatial learning axes.** No clear separation is seen between the child comorbid groups.
(MOV)
